# Comparison between Imaging and Physiology in Guiding Coronary Revascularization: A Meta-Analysis

**DOI:** 10.3390/jcm13092504

**Published:** 2024-04-24

**Authors:** Riccardo Improta, Gianluca Di Pietro, Michele Giansanti, Francesco Bruno, Ovidio De Filippo, Marco Tocci, Riccardo Colantonio, Gennaro Sardella, Fabrizio D’Ascenzo, Massimo Mancone

**Affiliations:** 1 Department of Clinical, Internal, Anesthesiology and Cardiovascular Sciences, Umberto I Hospital, Sapienza University of Rome, Viale del Policlinico 155 (Emodinamica A, Ottavo Padiglione, II Piano), 00161 Rome, Italy; riccardo.improta@uniroma1.it (R.I.); gianluca.dipietro@uniroma1.it (G.D.P.); marcotocci1392@gmail.com (M.T.); riccolantonio@gmail.com (R.C.); rino.sardella@uniroma1.it (G.S.); 2 Department of Medical Science, Division of Cardiology, Molinette Hospital, Turin University, 10124 Turin, Italy; cescobruno@hotmail.it (F.B.); ovidio.defilippo@gmail.com (O.D.F.); fabrizio.dascenzo@gmail.com (F.D.)

**Keywords:** percutaneous coronary intervention, imaging-guided PCI, physiology-guided PCI, intermediate coronary artery lesions

## Abstract

**Background**: Percutaneous coronary intervention (PCI) is a widely used revascularization strategy for coronary artery disease. The choice between imaging-guided and physiology-guided PCI has been a subject of debate. This meta-analysis aims to systematically compare outcomes between imaging and physiology-guided PCI and management of intermediate coronary lesions (ICLs). **Methods**: A comprehensive literature search was conducted across major databases for studies published up to December 2023 following PRISMA guidelines. Seven eligible studies comparing imaging-guided and physiology-guided PCI were selected for the final analysis. Relevant outcome measures included major adverse cardiovascular events (MACE), target vessel revascularization (TVR), target vessel failure (TVF), and target lesion revascularization (TLR). Subgroup analysis was performed for ICLs. **Results**: A total of 5701 patients were included in the meta-analysis. After a mean follow-up of 2.1 years, imaging-guided PCI was associated with lower rates of TVR compared to physiology-guided PCI (OR 0.70, 95% CI 0.52–0.95, *p* = 0.02); concerning MACE, TVF, and TLR, no differences were found. When the analysis was restricted to studies considering ICLs management, there were no differences between the two techniques. Meta regression analysis did not show any impact of acute coronary syndromes (ACS) presentation on MACE and TVR. **Conclusions**: The findings suggest that imaging-guided PCI may reduce the need for future revascularization of the target vessel compared to the functional-guided approach, and this result was not influenced by ACS presentation. These results may have important implications for clinical practice, guiding interventional cardiologists in selecting the most appropriate guidance strategy.

## 1. Introduction

Percutaneous Coronary Intervention (PCI) is the mainstay of treatment for coronary atherosclerotic disease. Recent trial results have, however, shed light on a neutral impact of PCI in the setting of chronic coronary syndromes so that a more individualized and precise approach to coronary lesions is warranted. As the field of interventional cardiology has evolved, two fundamental strategies have emerged to guide the decision-making process after, during, and post-PCI: intracoronary imaging and physiology-guided interventions. Flow Fractional Reserve (FFR), IntraVascular UltraSound (IVUS), and Optical Coherence Tomography (OCT) have their advantages and pitfalls and have been extensively validated for improving outcomes from indication to PCI optimization with respect to coronary angiography alone, as highlighted by evidence-based guidelines [1,2,3,4]. Treatment or deferral of intermediate coronary lesions (ICLs) based upon coronary physiology indices is a well-established approach supported by a variety of studies and current guidelines [3,4]; concerning post-interventional assessment, low post-PCI physiology values have been linked to adverse clinical events and cut-offs have been defined as a target to achieve during PCI to improve outcomes [1,5,6]. Intracoronary imaging may provide quantitative and qualitative data about intermediate coronary lesions, such as the thickness of the atheroma’s cap, plaque burden, and lipid content: these and other morphological high-risk features are typical of culprit lesions in Acute Coronary Syndromes (ACS) and add prognostic value over the FFR attribute alone [7,8]. For PCI optimization, with respect to physiology data, intracoronary imaging can show stent malapposition, stent edge dissections, and inappropriate landing zones; all of these characteristics have been proven to be associated with worse device-related outcomes [2]. Both technologies are currently widely used, and the choice of guidance modality should align with the operator’s expertise and institutional capabilities. Consequently, we performed this meta-analysis to compare imaging and physiology-guided percutaneous revascularization with a focus on intermediate coronary artery lesions (ICLs), given that a clear superiority between the two approaches has not yet been demonstrated.

## 2. Methods

### 2.1. Eligibility Criteria, Databases, and Search Strategy

The analysis was performed following the Preferred Reporting Items for Systematic Reviews and Meta-analyses (PRISMA) guidelines [9] and registered on PROSPERO (CRD42023474680). The following online databases were evaluated for articles published by December 2023: PubMed/MEDLINE, CENTRAL/CCTR (Cochrane Controlled Trials Register), Google Scholar, Scopus, and references from relevant articles. The subsequent terms, following a PICO strategy (population, intervention, control, outcomes), were searched in different combinations: “intracoronary imaging”, “coronary physiology”, “FFR”, “OCT”, “IVUS”, “PCI”, “outcomes”.

For the final inclusion in the analysis, titles of records were identified through database search, followed by the removal of duplicates. Abstracts were selected and, after analysis of full texts, when available, screened for eligibility.

**Figure 1:**
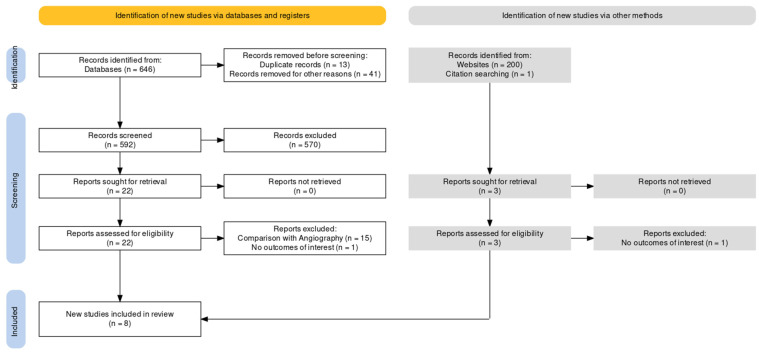
PRISMA flowchart for studies search.

Studies were comprehended if the following inclusion criteria were fulfilled: (1) two populations underwent FFR or imaging-guided PCI, (2) Outcomes for each group were reported, including MACE (Major Adverse Cardiovascular Events), TVF (Target Vessel failure), TVR (Target Vessel Revascularization), TLR (Target Lesion Revascularization). The study design was deemed to be irrelevant. Study-defined endpoints were considered. A flow diagram [10] is reported in Figure 1. Ethical approval was not requested, and no language restriction was applied.

### 2.2. Assessment of Risk of Bias

The admitted studies underwent stratification for risk of bias through The Risk of Bias in Non-Randomized Studies of Interventions tool (ROBINS-I) [11] for non-randomized studies and the RoB2 tool [12] for randomized trials (RTs). A severity scale was used to identify, in each domain and in the overall analysis, low, moderate, and serious risk of bias; in the end, the studies and their characteristics were classified into mild, moderate, and serious risk of bias. Two independent reviewers assessed the risk for bias. When there was a disagreement, a third reviewer made the final decision.

### 2.3. Statistical Analysis

Data inference was carried out only if at least one event in each group was found. Statistical heterogeneity was assessed through the inconsistency index I^2^ and tested for significance. High heterogeneity was defined for I^2^ indices over 50%. A random-effect model was used to pool data from the chosen studies if the test for heterogeneity was positive (*p* < 0.05), and a fixed-effect model was used in all other cases. A funnel plot was generated for each outcome and tested through Egger’s test to assess publication bias; in case of significant publication bias, outlier studies will be excluded from the main analysis in a stepwise fashion, and repeated testing will prove the bias resolution. Odds ratio with 95% confidence intervals (CIs) were calculated for the effect size for binary outcomes, and Forest plots were used to represent differences in clinical endpoints. A secondary analysis was performed for studies that only included ICLs and their revascularization in the study flowchart based on findings obtained by intracoronary imaging or physiology measurements. Meta-regression analysis was performed to investigate the role of ACS presentation as a moderator for effect size if there were enough data for at least 4 studies. A significant cut-off value of less than 0.05 was chosen to identify statistical relevance. All analyses were performed with Revman v5.4 and SPSS v29.0.

## 3. Results

### 3.1. Study Selection and Characteristics

Regarding the comparison of outcomes between functional and imaging-guided PCI, eight studies [13,14,15,16,17,18,19,20] were considered eligible for the analysis: two out of eight were randomized trials, while three had matched populations. Four trials included only patients with intermediate coronary artery lesions randomized to either revascularization or medical therapy based on FFR or imaging parameters; a secondary analysis comprehending only this subgroup of patients was performed. The average time of follow-up was 2.1 years, with only one study reporting very long-term outcomes (6 years); in ICLs studies, outcomes were recorded at a mean of 1.5 years. The studies’ characteristics are resumed in Table 1.

The analyzed population consisted of 2510 patients (of whom 1497 had ICLs) who underwent functional-guided PCI and 3191 (of whom 1512 had ICLs) with an imaging-guided PCI. Patients were male in 67% of cases with a median age of 65 (interquartile range 63.7–66.5), and 39% presented with ACS. The most frequent vessel treated was the left anterior descending artery (LAD), and 26% of patients had diabetes. Patient characteristics from each study are listed in Table 2.

The inclusion and exclusion criteria of selected studies, along with cut-offs for the treatment of intermediate coronary lesions, are gathered in Supplementary Tables S1 and S2. Overall, intermediate coronary lesion was defined from angiography estimation of a percentage of diameter stenosis between 40% and 70%. FFR cut-offs for revascularization were consistently homogenous (0.80 for three studies, 0.75 for one study), and imaging criteria for revascularization relied mostly on Minimum Lumen Areas (MLAs), varying from 3 mm^2^ to 4 mm^2^ and on plaque burden.

Qualitative assessment for bias of the studies with ROBINS-I and RoB2 tools is shown in Supplementary Figure S1. Even when propensity score matching was performed, we cannot exclude that some confounders may have not been considered so that every study has at least a moderate risk for confounding bias. Funnel plots and Egger’s testing for publication bias are reported in Supplementary Figures S2–S5, resulting in absence of publication bias.

### 3.2. Clinical Outcomes

The definition of clinical outcomes varied between studies, especially for composite endpoints (MACE). In the study by Koo et al. [16], MACE were chosen according to VARC-2 endpoint classification for coronary trials to follow the device-oriented composite endpoint definition. On the other hand, TVR, TVF, and TLR definitions were consistently similar among studies.

**Figure 2:**
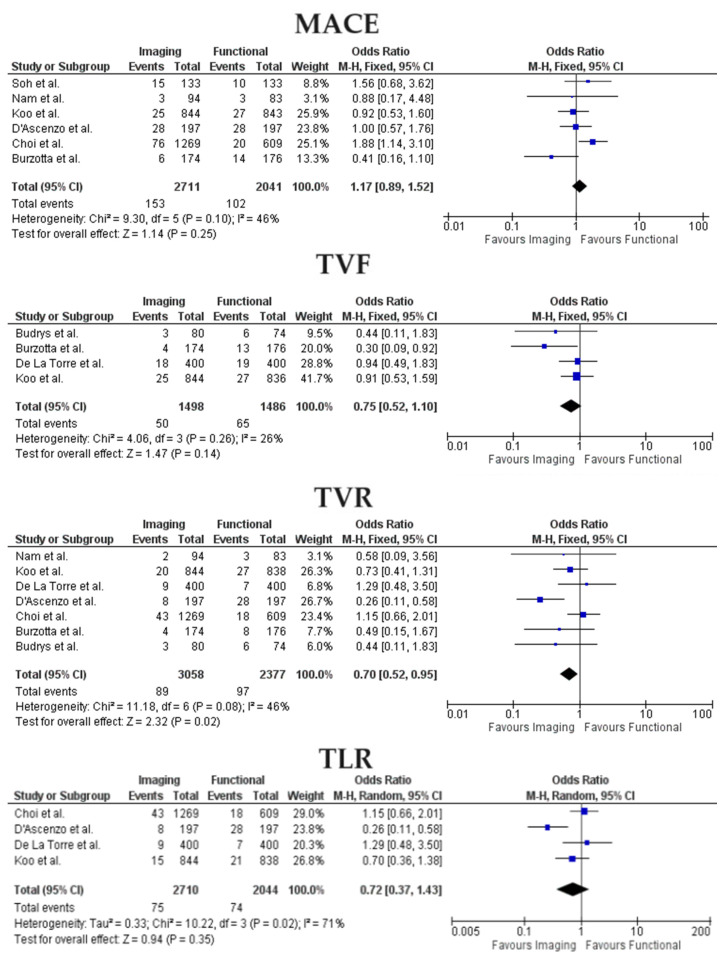
Forest Plots for imaging-guided vs functional-guided PCI. MACE, Major Adverse Cardiac Events; TVF, Target Vessel Failure; TVR, Target Vessel Revascularization; TLR; Target Lesion Revascularization [13,14,15,16,17,18,19,20].

Forest Plots for outcomes between imaging and functional-guided PCI are represented in Figure 2. Heterogeneity was overall low or moderate; only in the all-studies analysis I^2^ was significantly higher for the TLR outcome (71%), so a random-effect model was adopted.

Among imaging and functional-guided PCI, no statistically significant difference was found for the probability of MACE (OR 1.17, 95% CI 0.89–1.52, *p* = 0.25), TVF (OR 0.75, 95% CI 0.52–1.10, *p* = 0.14) and TLR (OR 0.72, 95% CI 0.37–1.43, *p* = 0.35) between the two groups. TVR rates were lower in the imaging-PCI group (OR 0.70, 95% CI 0.52–0.95, *p* = 0.02).

**Figure 3:**
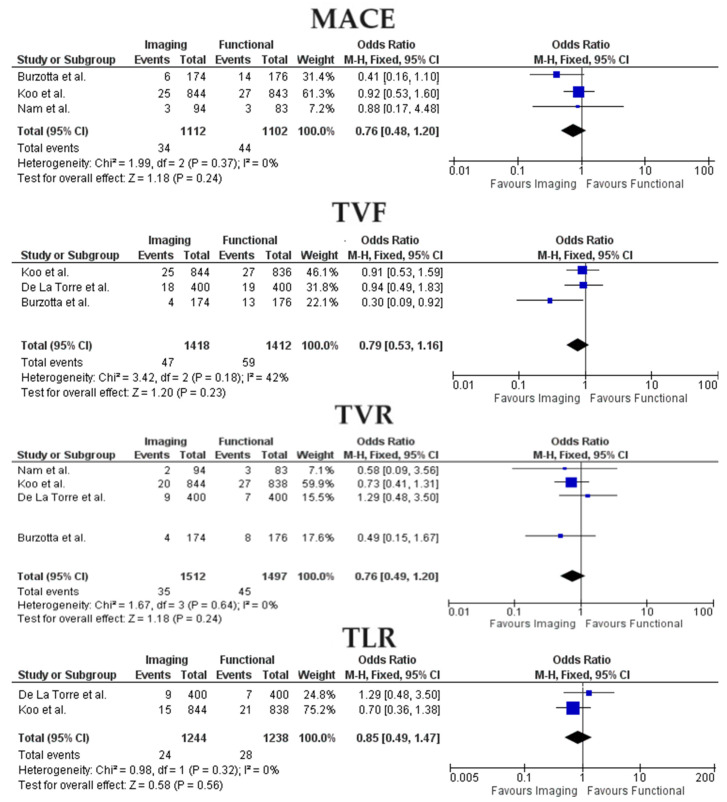
Forest Plots for imaging-guided vs functional-guided PCI. MACE, Major Adverse Cardiac Events; TVF, Target Vessel Failure; TVR, Target vessel Revascularization; TLR, Target Lesion Revascularization [13,14,16,19].

When restricting the analysis to studies that only comprehended ICLs, the advantage in terms of TVR incidence for the imaging group was lost (OR 0.76, 95% CI 0.49–1.20, *p* = 0.24) while the absence of differences concerning MACE (OR 0.76, 95% CI 0.48–1.20, *p* = 0.24), TVF(OR 0.79, 95% CI 0.53–1.16, *p* = 0.23) and TLR (OR 0.85, 95% CI 0.49–1.47, *p* = 0.56) was confirmed as shown in Figure 3.

Meta regression analysis to evaluate the role of ACS presentation as a moderator for MACE and TVR effect size between imaging and physiology-guided PCI showed no statistically significant impact (Bubble plots are reported in Supplementary Figure S6).

## 4. Discussion

The main findings from our study can be summarized as follows:

1. Between functional and imaging-guided PCI, there were no differences in rates of MACE, TVF, and TLR;
2. The incidence of TVR was lower in the imaging-PCI group;
3. When considering only studies focused on ICLs management, no significant differences were found between the two analyzed populations concerning MACE, TVR, and TVF;
4. Presentation with acute coronary syndrome was not a significant moderator for MACE and TVR across the two groups.

The primary finding of our meta-analysis was the significant decrease in target vessel revascularization in the imaging-guided PCI group when compared to the physiology-guided PCI group. This result is in accordance with several studies evaluating the effectiveness of intracoronary imaging [21,22,23,24] and supports the concept that imaging-guided PCI techniques can help improve stent placement and post-procedural results, potentially reducing the likelihood of restenosis and the need for repeat revascularization with respect to physiology optimization. For instance, even when optimal post-PCI FFR values are achieved [25], subtle stent underexpansion and malapposition, geographical miss, and angiographically silent edge dissection can lead to in-stent restenosis and worse prognosis [26,27].

Comparisons between the two approaches have already been performed without demonstrating a clear superiority [28,29]: this might be explained because some physiological and imaging features to determine critical lesions and sub-optimal PCI results are correlated, such as low FFR values and small minimum areas [30,31].

In a network meta-analysis (NMA) comparing angiography, physiology, and imaging in guiding PCI [28], IVUS resulted in lower stent thrombosis rates compared to FFR. Even in the most recent NMA by Kuno and colleagues [29], both intravascular imaging and functional-guided PCI were associated with reduced risk of MACE and MI compared to sole angiography, but stent thrombosis and TLR were significantly reduced only by IVI guidance; moreover, intravascular imaging-guided PCI ranked first concerning MACE, cardiovascular death, stent thrombosis, and TLR. They found no differences among clinical presentations (acute or chronic coronary syndromes). After a meta-analysis evaluating the accuracy of imaging-derived MLA in predicting functionally significant lesions [32], the authors concluded that MLA and MLD have a moderate correlation with FFR findings: especially for left main disease, an imaging approach could safely replace functional evaluation. A deeper-than-expected link might indeed exist between atherosclerosis burden and hemodynamic changes: while the plaque keeps growing in low-shear stress regions, the lumen reduction alters the local hemodynamic forces, leading to increased plaque vulnerability and impaired physiology indices [33,34]. Teleman et al., in the FFR-REACT trial [35], evaluated an IVUS optimization strategy against a standard approach in post-PCI lesions with an FFR value of less than 0.9. Although no differences in Device Oriented Composite Endpoints (DOCE) were observed, probably because of the lack of events, a trend for less TVR and significantly increased FFR and MLA values was found in the imaging-guided optimization. A recent meta-analysis by Sanchez et al. comparing FFR-guided and non-physiology-guided PCI demonstrated reduced all-cause mortality and MI risk in the first group, but it also included studies where the control group was represented by a sole angiography [36].

In our ICLs sub-analysis, rates of vessel-oriented outcomes were similar between the two groups: this could be explained by the reduction of the analyzed population and subsequent loss of power to draw significant conclusions. However, it has already been proven that in FFR-negative lesions, low thickness of cap atheroma significantly predicted adverse events; furthermore, this group presented significantly more vulnerable OCT characteristics than FFR-negative/thick atheroma cap lesions, suggesting that vascular imaging should be the preferred approach in the evaluation of intermediate coronary lesions revascularization [7]. This concept is also supported by intracoronary imaging studies evaluating predictors of plaque vulnerability and ACS [37,38,39]. When performing intravascular imaging to determine the significance of an intermediate coronary artery lesion, the leading plaque characteristic guiding operators in the decision-making process is the MLA. In the meta-analysis by D’Ascenzo and colleagues [32], pooled MLA thresholds to identify functionally significant lesions were lower than the currently prognostic adopted cut-offs (IVUS MLA 2.8 mm^2^ for vessels with a reference diameter more than 3 mm while the OCT MLA was 1.96 mm^2^). This finding supports the concept that the functional indices only represent one of a spectrum of clinically significant or prognostic plaque characteristics that may guide coronary revascularization. Imaging-guided PCI could result in “overtreatment” of coronary lesions compared to physiology-guided PCI because of the greater amount of plaque characteristics analyzed. Whether this approach to intermediate coronary lesions translates into improved outcomes remains a matter of debate and worthy of dedicated trials. The recently presented results of the PREVENT trial [40] are encouraging in recommending preventive stenting of FFR-negative intermediate coronary artery lesions with imaging vulnerability characteristics: in the trial, patients randomized to the prophylactic group had an 89% lower risk of the composite primary endpoint of cardiac death, target-vessel MI, ischemia-driven target vessel revascularization, or hospitalization for unstable or progressive angina at 2 years compared with those in the OMT group.

Acute coronary syndromes represent a specific subset in which intracoronary imaging PCI guidance could be of valuable support. First, in ACS, non-culprit lesions often present vulnerability characteristics explaining the increased rates of non-culprit related events in these patients due to the patient’s higher burden of cardiovascular risk factors and coronary inflammation [41]. Secondly, there is a paucity of data regarding physiology indices PCI guidance in acute coronary syndromes, especially for STEMI patients; the microcirculation and vascular bed modifications occurring during an ACS might alter the functional evaluations [42]. Even if other studies [43] already demonstrated the favorable outcomes in imaging-guided PCI along with an increased use [44] of intravascular imaging in acute patients, in our analysis, the percentage of ACS was not a significant moderator for TVR and MACE between the two groups: this might be explained for the different imaging modality used in the included manuscripts (OCT and IVUS might have proper advantages and disadvantages) or for the insufficiency of data available from all the studies to draw definite conclusions. In the ongoing OCT-CONTACT study [45], intermediate non-culprit coronary lesions in a post-primary PCI setting in STEMI patients are randomized to OCT-guided PCI or standard of care: this will shed light on the impact of imaging-guided vulnerable plaque stenting even in this subset of patients. Vulnerability criteria include MLA, fibroatheroma cap thickness, and ruptured plaque.

The reduction in target vessel revascularization observed in the imaging-guided PCI group has significant clinical implications. Fewer repeat revascularization procedures not only improve patient outcomes but also decrease the economic burden associated with healthcare costs. Moreover, reduced target vessel revascularization is indicative of improved long-term patency of coronary stents and better management of coronary artery disease, ultimately leading to a better quality of life for patients. Even if FFR was associated with a favorable cost-efficacy profile for reducing unnecessary PCI [46], an imaging-optimized angioplasty and careful evaluation of all the vessel lesions characteristics could reduce the need for future revascularization in an urgent or emergent setting. In a recent study about the cost-effectiveness of imaging-guided PCI, despite initial increased costs associated with devices and procedural tools, at longer-term follow-ups was shown to be more cost-effective than angiography-guided PCI, along with demonstrating an increasing quality of life [47]. On the other hand, other factors, such as procedural time, radiation exposure, and resource availability, should also be considered when making clinical decisions. Of course, not every coronary angioplasty should undergo imaging assessment when presenting with features of low complexity from a cost-optimization perspective. Complex PCI includes a variety of definitions but represents the ideal setting for intravascular imaging use: the recent results of the RENOVATE COMPLEX PCI trial [47] concluded that intravascular imaging-guided PCI led to a lower risk of composite endpoint of cardiac death, target-vessel–related myocardial infarction, or clinically driven target-vessel revascularization than angiography-guided PCI. Further research is needed to explore the long-term clinical outcomes, cost-effectiveness, and patient-reported outcomes associated with imaging-guided versus physiology-guided PCI.

## 5. Limitations

It is important to acknowledge the heterogeneity among the included studies in this meta-analysis: variability in study designs, patient populations, and the specific imaging or physiology guidance techniques used may have influenced the results. Definitions of MACE varied between selected studies. The impact of clinical and procedural characteristics on treatment effects was hindered by the absence of patient-level data. Finally, as already mentioned, the limited number of studies in the sub-analysis for ICLs could possibly have reduced our power to draw definite conclusions. We preferred not to perform sensitivity analysis for randomized controlled trials because the resulting analysis could have been significantly underpowered due to the paucity of data.

## 6. Future Directions

The rapidly evolving landscape of cardiovascular imaging and functional-guided PCI holds promising avenues for future exploration and innovation. Continued advancements in imaging modalities will enable more precise lesion characterization. Integration of artificial intelligence and machine learning algorithms into these imaging platforms may revolutionize lesion assessment, risk prediction, and treatment planning. Additionally, the integration between functional and imaging assessments may provide a comprehensive approach to guide PCI interventions based on both anatomical and physiological considerations and could pave the way for personalized and minimally invasive interventions. Further research is warranted to establish the long-term outcomes and cost-effectiveness of the single and integrated approaches in different subsets of patients (complex coronary interventions, acute coronary syndromes) to understand the strategy ensuring greater benefits for the patient.

## 7. Conclusions

Among patients undergoing PCI for CAD, imaging-guided PCI reduced target vessel revascularization rates when compared to a physiology-guided approach. Differences in MACE, TVF, and TLR were not significantly affected by the adopted strategy. ACS presentation did not impact as a moderator for MACE and TVR risk. When restricting the analysis to studies evaluating only ICLs, no differences were observed between the two techniques concerning MACE, TVF, and TVR.

## Figures and Tables

**Table 1:** Studies Characteristics. N, Number; NA, Non Available; RCT, Randomized Controlled Trial; PSM, Propensity Score Matched; IVUS, Intra-Vascular Ultra-Sonography, OCT, Optical Coherence Tomography; FFR, Flow Fractional Reserve.

Study	Publication Year/Enrollment Year	Country	Study Design	Patients (N)			Imaging Type
				Total	Imaging	FFR	
Burzotta et al. [13]	2020/2013–2019	Italy	RCT	350	174	176	OCT
Nam et al. [19]	2010/2006–2008	Korea	Observational	167	83	94	IVUS
D’Ascenzo et al. [17]	2017/2009–2015	Italy, France	PSM	394	197	197	OCT
De La Torre et al. [14]	2013/NA	Spain	PSM	800	400	400	IVUS
Koo et al. [16]	2022/2016–2022	China, Korea	RCT	1682	844	838	IVUS
Soh et al. [15]	2023/2014–2015	China	PSM	266	133	133	IVUS/OCT
Budrys et al. [18]	2023/NA	Lithuania	Observational	154	80	74	IVUS
Choi et al. [20]	2023/NA	Korea	Observational	1878	1269	609	IVUS

**Table 2:** Population characteristics for single studies. Values significantly different from the original studies are highlighted in bold. N, Number; SD, Standard Deviation; FFR, Flow Fractional Reserve; PCI, Percutaneous coronary Intervention; CABG, Coronary Artery Bypass Grafting; SHID, stale ischemic heart disease; ACS, Acute coronary Syndrome; LAD, Left Anterior Descending.

Study	Age- FFR/Imaging (Years ± SD)	Male- FFR/Imaging (%)	Diabetes- FFR/Imaging (%)	Previous PCI- FFR/Imaging (%)	LAD- FFR/Imaging (%)	SIHD- FFR/Imaging (%)	ACS- FFR/Imaging (%)	Multivessel Disease- FFR/Imaging (%)
Burzotta et al. [13]	68 ± 10/69 ± 9	71.6/77.6	34.7/36.2	41.5/43.7	66.7/60.6	79/82.2	21/17.8	53.3/47.7
Nam et al. [19]	63 ± 9/62 ± 9	66.3/58.2	21.7/22.5	20.5/12.8	48.2/58.2	45.8/36.2	54.2/63.8	66.3/51.1
D’Ascenzo et al. [17]	64 ± 11/ 63 ± 10	76.6/77.7	24.4/19.3	N/A	56.9/64.5	N/A	N/A	16.2/21.8
De La Torre et al. [14]	65.9 ± 9.5/ 65.2 ± 10	74.2/74.5	39.8/37.5	23.3/19	57.2/57.4	31.5/30	N/A	22.5/20.2
Koo et al. [16]	65.4 ± 9.4/ 64.8 ± 9.9	69.7/71.4	32.5/33.4	19.7/19.3	62.4/61.5	61.9/64.5	30.1/28.9	53.1/50.9
Soh et al. [15]	N/A	N/A	N/A	N/A	N/A	N/A	N/A	N/A
Budrys et al. [18]	66.3 ± 9.2/ 66.2 ± 9	73/71	21.6/18.8	**39.2/57.5**	82.4/82.5	74.3/75	N/A	85.1/85
Choi et al. [20]	N/A	N/A	N/A	N/A	N/A	N/A	N/A	N/A

## Data Availability

The data underlying this article are available in the article and in its online Supplementary Material.

## Supplementary Materials

The following supporting information can be downloaded at: https://www.mdpi.com/article/10.3390/jcm13092504/s1. Table S1: Inclusion and Exclusion criteria from included studies, Table S2: Imaging and physiology criteria for ICLs revascularization, Figure S1: Bias Assessment, Figure S2: Publication Bias Assessment for MACE, Figure S3: Publication Bias Assessment for TVR, Figure S4: Publication Bias Assessment for TVF, Figure S5: Publication Bias Assessment for TLR, Figure S6: Bubble Plots from metaregression analysis.
