# Supplementary material for: Comparison between Imaging and Physiology in Guiding Coronary Revascularization: A Meta-Analysis
Source: J Clin Med. 2024 Apr 24;13(9):2504. doi: 10.3390/jcm13092504 (PMC11084876; doi:10.3390/jcm13092504)
Supplement: Supplementary file 1 [file jcm-13-02504-s001.zip › jcm-2960644-supplementary.pdf]

Supplementary Material

- **TableS1: Inclusion and exclusion criteria of single studies.**
- **TableS2: Imaging and physiology criteria for Intermediate Coronary Artery Lesions (ICLs) revascularization**
- **FigureS1: Bias Qualitative Assessment**
- **FigureS2: Publication Bias Assessment for MACE**
- **FigureS3: Publication Bias Assessment for TVR**
- **FigureS4: Publication Bias Assessment for TVF**
- **FigureS5: Publication Bias Assessment for TLR**
- **FigureS6: Bubble plots from Meta-Regression analysis**

**Study**	**Inclusion criteria**	**Exclusion criteria**
Burzotta et al.	- ICL defined as 40% to 80% stenosis by visual assessment in a non-distal segment of a major epicardial coronary artery.<br>- Single vessel disease<br>- Multivessel disease including only ICLs.<br>- Multivessel disease including at least one ICL and previously treated angiographic critical stenosis	- Age≤18 years<br>- Impossibility to give informed consent<br>- Female sex with child-bearing potential<br>- Life expectancy≤12 months<br>- Factors making clinical follow-up difficult<br>- LVEF<30%<br>- Recent (<7 days) STEMI<br>- Recent (<48 hours) NSTEMI<br>- Prior STEMI in the territory supplied by the vessel under investigation<br>- Severe myocardial hypertrophy<br>- Severe valvular heart disease<br>- Significant platelet count alteration (<100.000 cells/mm^3 or >700.000 cells/mm^3)<br>- Gastrointestinal bleeding requiring surgery or blood transfusions within 4 previous weeks<br>- History of clotting pathology<br>- Known hypersensitivity to aspirin, heparin, contrast dye

		- Advance CKD with GFR<30 ml/min
Nam et al.	ICL defined as 40% to 70% stenosis by visual assessment in a proximal or mid part of a major epicardial coronary artery (no ischemia test needed)	• Primary or emergency setting PCI for ACS.<br>• Prior CABG.<br>• Multiple lesions on the same vessel.<br>• Left main disease, primary myocardial disease or any major life threatening illness.<br>• Contraindications to adenosine, ASA and clopidogrel.
D'Ascenzo et al.	Patients treated for ACS using OCT (from January 2014 to October 2015) or FFR (from January 2009 to December 2012) on culprit or non-culprit lesions.	- Patients with poor image quality, incomplete pull-back or missingdata.<br>- Absence of signedinformed consent<br>- Patients who did not implant II generation drug eluting stent,<br>- Age<18 years old, pregnancy or shock, incomplete clinical data or loss at follow-up
De La Torre et al.	De novo ICL	LMA lesions
Koo et al.	De novo ICL (40 to 70%) in a target vessel with visual estimation diameter≥2,5mm.	- Life expectancy < 2 years.<br>- Target lesion in LMA or previous CABG.<br>- Increased bleeding risk
Soh et al.	NA	NA
Budrys et al.	Hemodynamically significant (FFR <0.8) long lesions necessitating > 30 mm of stent in patients with NSTEMI or Stable coronary artery disease	
Choi et al.	Successful PCI and High clinical/lesion/procedure related factor	-Cardiogenic shock at the index admission<br>-Subject treated with only BMS or plain old balloon angioplasty during the index procedure.<br>- Patients who are actively participating in another drug or device investigational study, which have not completed the primary endpoint follow-up period.

Table S1. Inclusion and exclusion criteria of single studies. NA, Not Available; FFR, Flow Fractional Reserve; PCI, Percutaneous coronary Intervention; CABG, Coronary Artery Bypass Grafting; ACS, Acute coronary Syndrome; LAD, Left Anteriore

Descending;LMA, Left Main Artery; IVUS, Intra-Vascular Ultra-Sonography, OCT, Optical Coherence Tomography; CKD, Chronic Kidney Disease; GFR, Glomerular Filtration Rate.

**Study**	**FFR**	**Imaging**
Burzotta et al.	FFR≤0,80	- Area Stenosis >75%.<br>- Area Stenosis between 50% and 75% with MLA<2,5mm^2 or plaque rupture.
Nam et al.	FFR<0,8	MLA<4mm^2
De La Torre et al.	FFR<0,75	Lesions with plaque burden>50% and:<br>- MLA<4mm^2 in vessels with reference diameter >3mm.<br>- MLA<3,5mm^2 in vessels with diameter between 2,5mm and 3mm.
Koo et al.	FFR<0,8	- MLA<3mm^2<br>- MLA 3-4mm^2 with plaque burden>70%

Table S2. Imaging and physiology criteria for Intermediate Coronary Artery Lesions (ICLs) revascularization. FFR, Flow Fractional Reserve;MLA, Minimal Lumen Area.

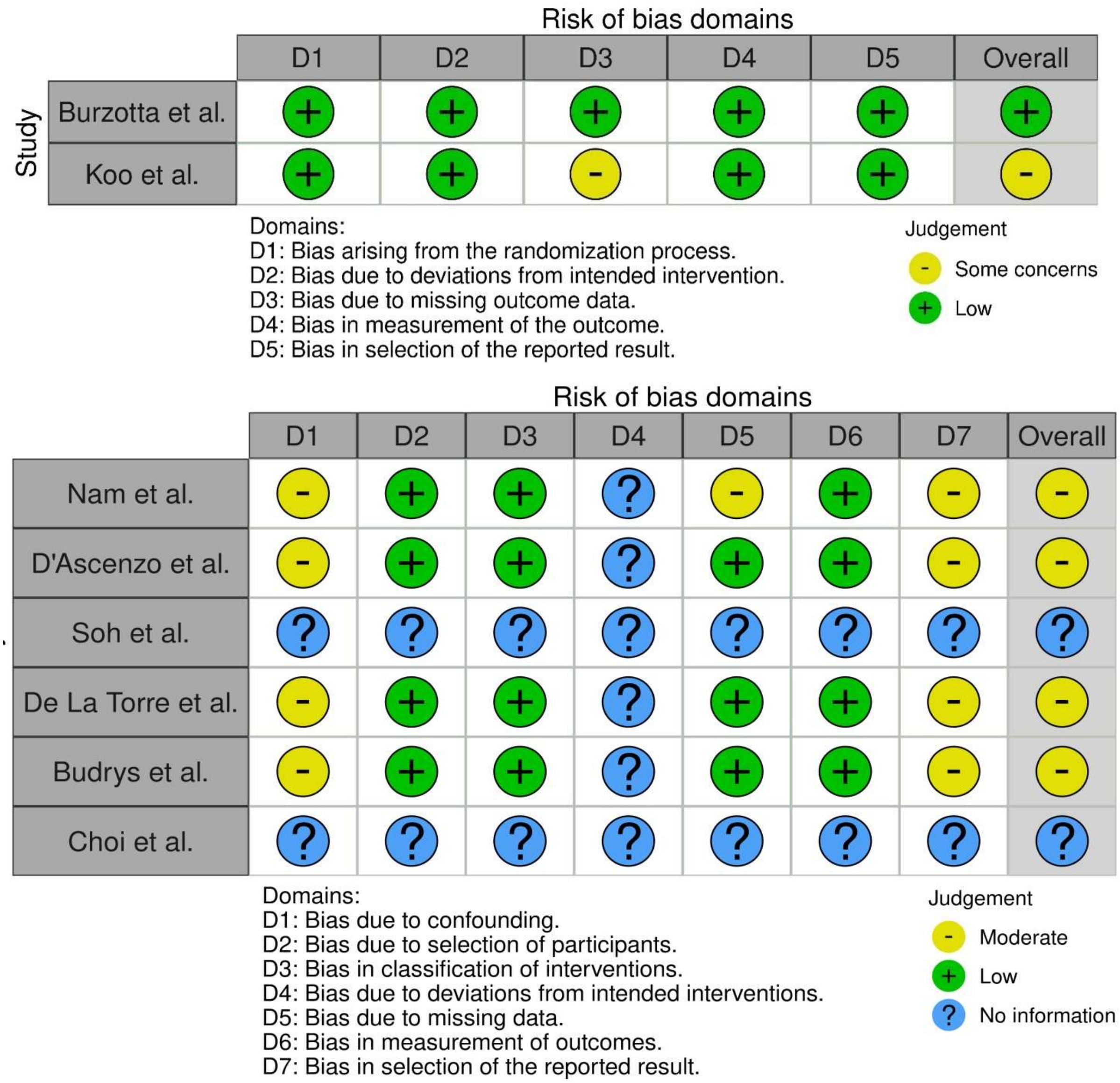

Figure S1. Bias Qualitative Assessment. In the upper part analysis for randomized controlled trials; in the lower part analysis for observational studies.

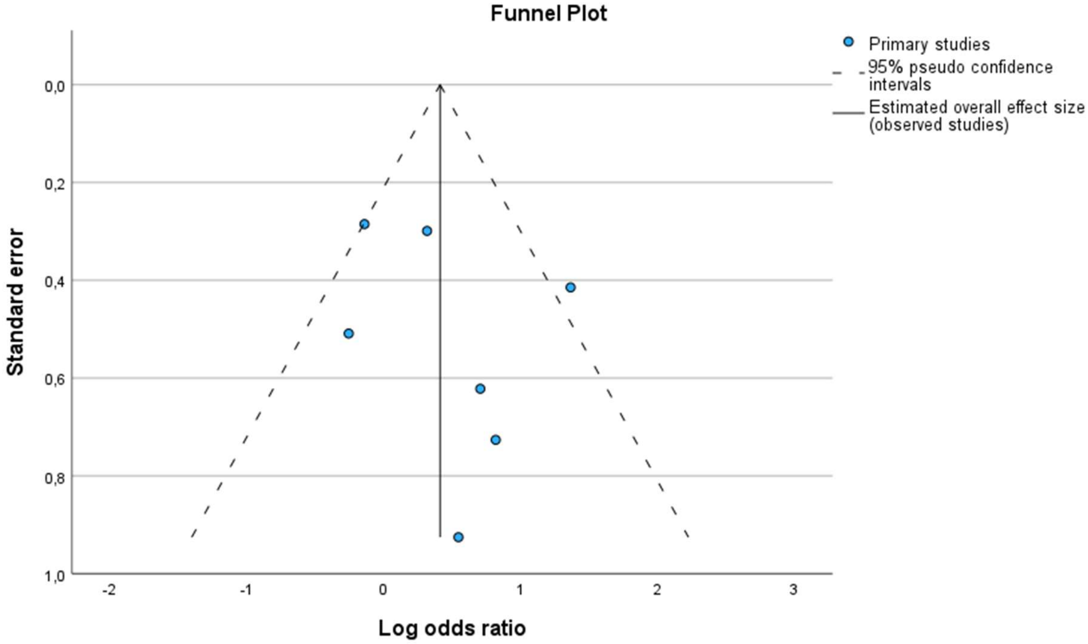

Egger's Regression-Based Test[a]

Parameter	Coefficient	Std. Error	t	Sig. (2-tailed)	95% Confidence Interval Lower	95% Confidence Interval Upper
(Intercept)	-,576	,5433	-1,061	,349	-2,085	,932
SE[b]	1,386	1,4408	,962	,391	-2,615	5,386

a. Random-effects meta-regression

b. Standard error of effect size

FigureS2. Publication Bias Assessment for MACE.

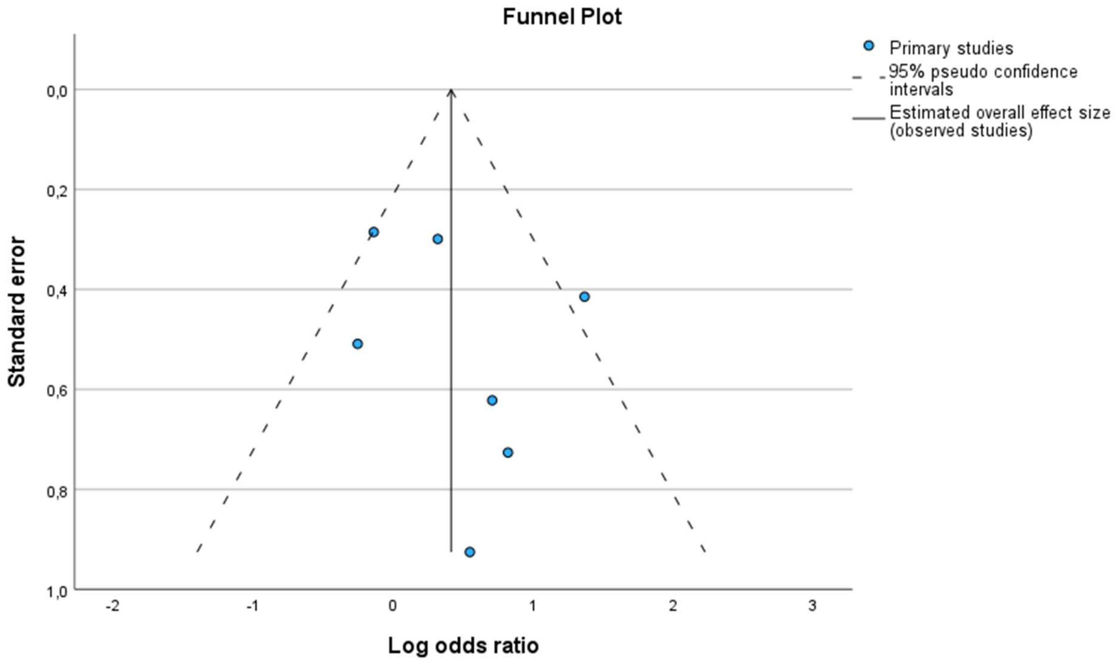

Egger's Regression-Based Test[a]

Parameter	Coefficient	Std. Error	t	Sig. (2-tailed)	95% Confidence Interval Lower	Upper
(Intercept)	,010	,6701	,014	,989	-1,713	1,732
SE[b]	,887	1,3605	,652	,543	-2,610	4,384

a. Random-effects meta-regression

b. Standard error of effect size

FigureS3. Publication Bias Assessment for TVR.

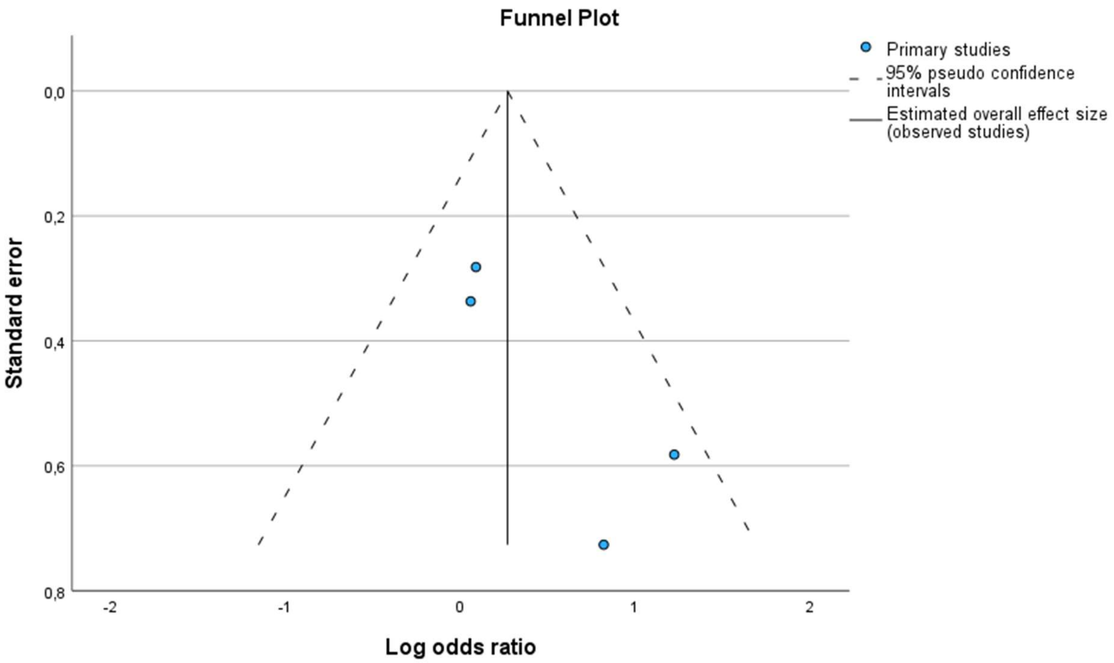

Egger's Regression-Based Test[a]

Parameter	Coefficient	Std. Error	t	Sig. (2-tailed)	95% Confidence Interval Lower	95% Confidence Interval Upper
(Intercept)	-,680	,5632	-1,207	,351	-3,103	1,744
SE[b]	2,558	1,4427	1,773	,218	-3,649	8,765

a. Random-effects meta-regression

b. Standard error of effect size

FigureS4. Publication Bias Assessment for TVF.

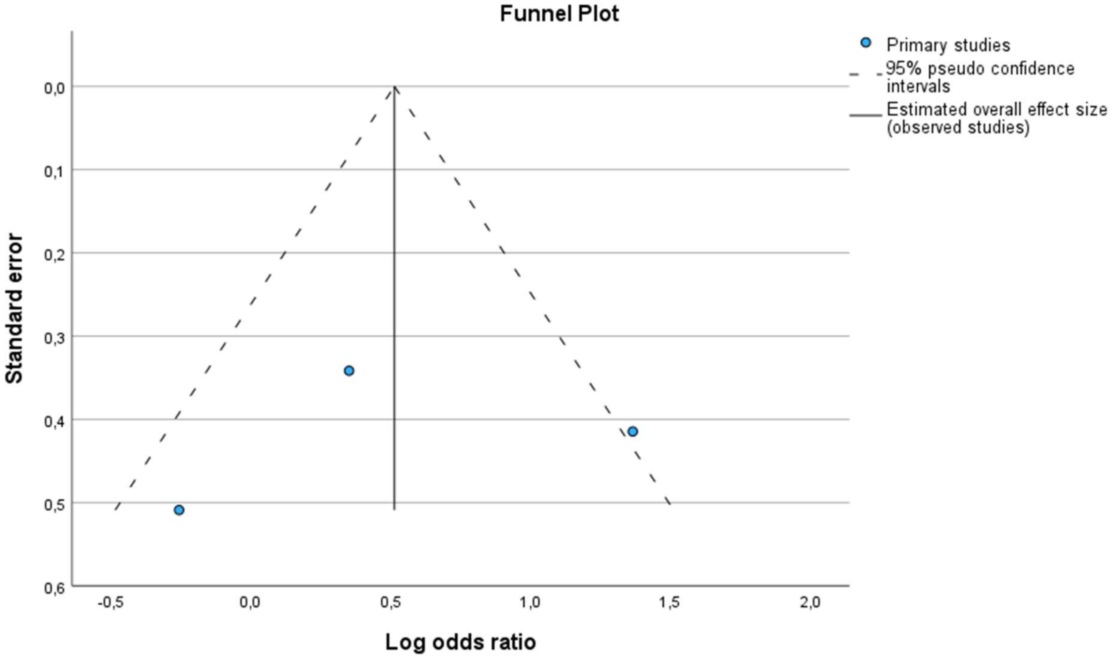

Egger's Regression-Based Test[a]

Parameter	Coefficient	Std. Error	t	Sig. (2-tailed)	95% Confidence Interval Lower	95% Confidence Interval Upper
(Intercept)	2,156	3,7531	,574	,668	-45,532	49,843
SE[b]	-3,954	8,8591	-,446	,733	-116,518	108,611

a. Random-effects meta-regression

b. Standard error of effect size

FigureS5. Publication Bias Assessment for TLR.

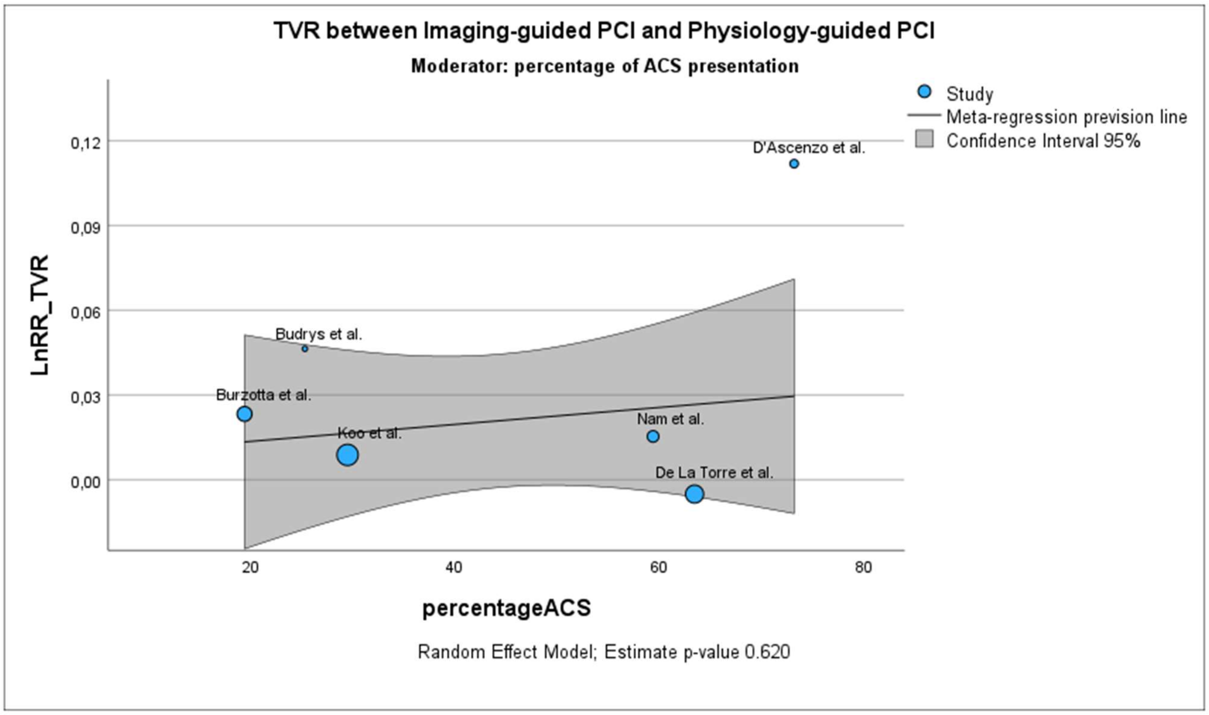

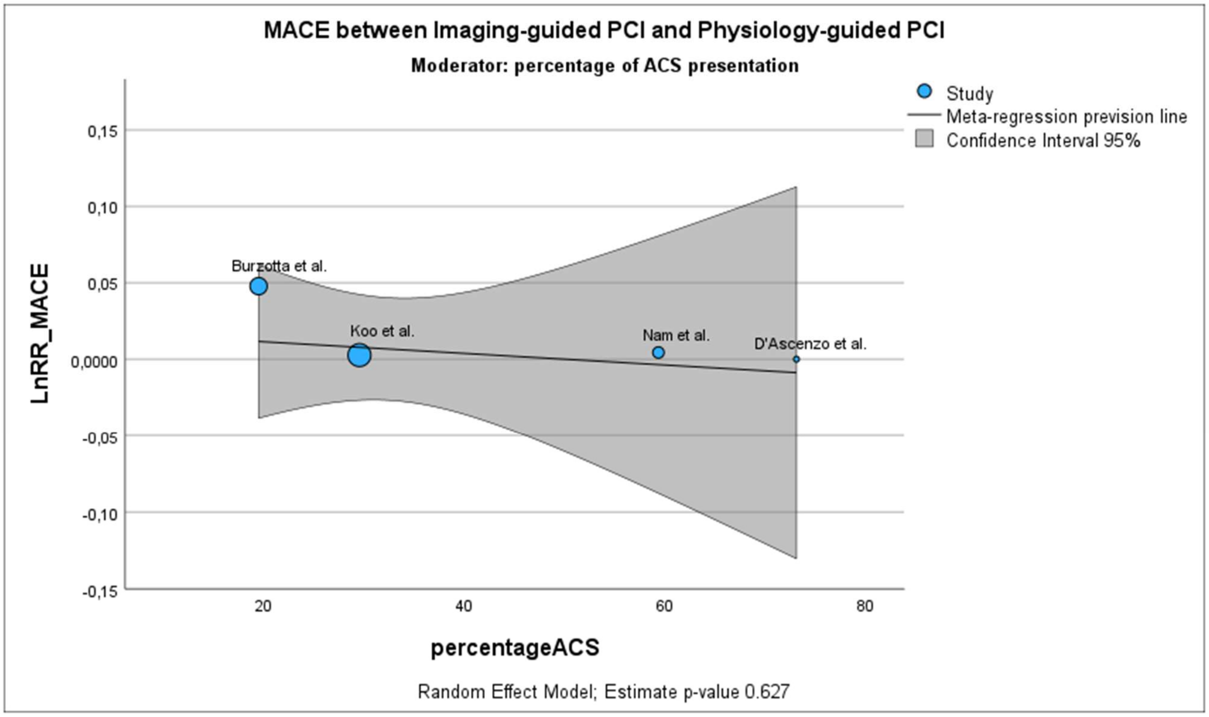

FigureS6. Bubble plots from Meta-Regression analysis. Upper part: Bubble plot for TVR between imaging and physiology guided PCI with ACS presentation as a moderator. Lower part: Bubble plot for MACE between imaging and physiology guided PCI with ACS presentation as a moderator. ACS, Acute coronary Syndrome; MACE, Major Adverse Cardiovascular Events; PCI, percutaneous coronary interventions;TVR, target vessel revascularization.
